# Physiological responses of two moss species to the combined stress of water deficit and elevated N deposition (II): Carbon and nitrogen metabolism

**DOI:** 10.1002/ece3.2521

**Published:** 2016-10-04

**Authors:** Bin‐yang Liu, Chun‐yi Lei, Jian‐hua Jin, Yi‐yun Guan, Shan Li, Yi‐shun Zhang, Wei‐qiu Liu

**Affiliations:** ^1^ Guangdong Key Laboratory of Plant Resources School of Life Sciences Sun Yat‐sen University Guangzhou China; ^2^ The State Key Laboratory of Vegetation and Environment Change Institute of Botany The Chinese Academy of Sciences Beijing China; ^3^ Heishiding Nature Reserve of Guangdong Province Zhaoqing China

**Keywords:** carbon metabolism, compensation effect, drought, moss, nitrogen, nitrogen assimilation, stress

## Abstract

Nitrogen (N) deposition levels and frequencies of extreme drought events are increasing globally. In efforts to improve understanding of plants' responses to associated stresses, we have investigated responses of mosses to drought under elevated nitrogen conditions. More specifically, we exposed *Pogonatum cirratum* subsp. *fuscatum* and *Hypnum plumaeforme* to various nitrate (KNO_3_) or ammonium (NH_4_Cl) treatments, with and without water deficit stress and monitored indices related to carbon (C) and N metabolism both immediately after the stress and after a short recovery period. The results show that N application stimulated both C and N assimilation activities, including ribulose‐1,5‐bisphosphate carboxylase, glutamine synthetase/glutamate synthase (GS/GOGAT), and glutamate dehydrogenase (GDH) activities, while water deficit inhibited C and N assimilation. The mosses could resist stress caused by excess N and water deficit by increasing their photorespiration activity and proline (Pro) contents. However, N supply increased their sensitivity to water stress, causing sharper reductions in C and N assimilation rates, and further increases in photorespiration and Pro contents, indicating more serious oxidative or osmotic stress in the mosses. In addition, there were interspecific differences in N assimilation pathways, as the GS/GOGAT and GDH pathways were the preferentially used ammonium assimilation pathways in *P. cirratum* and *H. plumaeforme* when stressed, respectively. After rehydration, both mosses exhibited overcompensation effects for most C and N assimilation activities, but when supplied with N, the activities were generally restored to previous levels (or less), indicating that N supply reduced their ability to recover from water deficit stress. In conclusion, mosses can tolerate a certain degree of water deficit stress and possess some resilience to environmental fluctuations, but elevated N deposition reduces their tolerance and ability to recover.

## 1 Introduction

Atmospheric nitrogen (N) deposition has risen sharply during recent decades and is expected to increase further in the near future (Fang, Gundersen, Mo, & Zhu, 2008; Galloway et al., 2008; Liu et al., 2013; Steven, Dise, Mountford, & Gowing, 2004). The land area affected by drought and the frequency of extreme drought events are also expected to increase during the 21st century.

Various studies have shown that excess N and drought can induce oxidative or osmotic stresses in plants and affect both their primary and secondary metabolism (Bauer et al., 2004; Fresneau, Ghashghaie, & Cornic, 2007; Guo, Schinner, Sattelmacher, & Hansen, 2005; Irigoyen, Einerich, & Sánchez‐Díaz,^1992^; Liu et al., 2015a; Pearce, Woodin, & Van der Wal, 2003). However, we still have little knowledge of the effects of elevated N on plants' responses to drought. Few studies have addressed these phenomena, and the results have been conflicting: Some have found that N deposition mitigated the adverse effects of drought on plants, while others have found the opposite (Betson et al., 2007; Friedrich et al., 2012; Liu et al., 2015a; Zhou, Zhang, Ji, Downing, & Serpe, 2011). The consequent lack of understanding of the combined effects of elevated N deposition and drought stress on plants is hindering the development of robust knowledge of plants' responses to shifts in precipitation and other climate changes under different N deposition regimes.

Further knowledge is also required of the “compensation effect,” that is, plants' typical positive response to stress or wounding (after removal of the stress factors), which results in their net productivity recovering or even exceeding that of unstressed or uninjured control plants (Belsky, 1986).

Mosses are valuable organisms for examining such responses as they are small, relatively primitive land plants that have no cuticle, so they are more sensitive to environmental changes than most plants (Harmens et al., 2011; Schröder et al., 2014). Excess N has distinct effects on both their primary and secondary metabolism, and generally results in increased levels of total N and numerous metabolites, including soluble proteins, arginine, aspartic acid, phenylpropanoids, triterpenes, and total alkaloids (Koranda, Kerschbaum, Wanek, Zechmeister, & Richter, 2007; Liu, Liu, Lei, Zhang, & Guo, 2011; Liu et al., 2015a; Paulissen, Besalú, de Bruijn, Van der Ven, & Bobbink, 2005). Furthermore, because mosses are poikilohydric, they are generally highly dependent on water, although a few species can resist a certain degree of drought via various mechanisms (Lou, 2006; Ruibal et al., 2012; Zhao, Shi, Liu, Jia, & Li, 2015). Several studies have addressed mosses' drought‐resistance mechanisms (Oliver, Veiten, & Wood, 2000; Ruibal et al., 2012; Zhao et al., 2015). However, little is known about their responses to simultaneous exposure to excess N and drought (Jones, Oxley, & Ashenden, 2002), and no published studies appear to have addressed their compensation effects following stress (if present). Clearly, therefore, further analysis of these responses, effects, and associated mechanisms is required to enable robust assessment of the fate of these plants in a changing world.

To improve knowledge of these phenomena, we have investigated primary metabolism, secondary metabolism, and hormone regulation responses of *Pogonatum cirratum* subsp. *fuscatum* and *Hypnum plumaeforme* (two moss species that are widely distributed in South China) to drought and subsequent short‐term recovery under varied nitrate (KNO_3_) or ammonium (NH_4_Cl) exposure treatments. We have previously reported results pertaining to secondary metabolism, including acclimation characters of the mosses to drought stress and interspecific differences in responses of their phenylpropanoid metabolism to water deficit (Liu et al., 2015a). In the study reported here, we focused on several primary metabolism processes. More specifically, we explored responses of the mosses' carbon and nitrogen metabolism process and interactions between associated metabolic pathways to combined water deficit and N application treatments, compensation effect in them following water deficit, and the physiological mechanism involved.

## 2 Materials and methods

### 2.1 Experimental design and stress treatment

**Figure 1:**
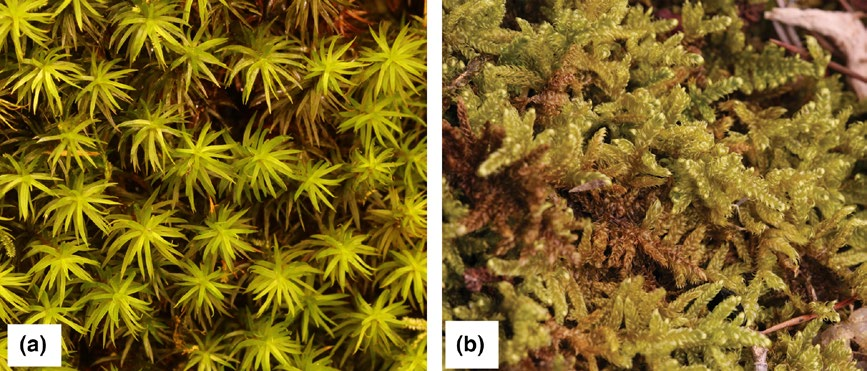
Moss species investigated in the study: *Pogonatum cirratum* subsp. *fuscatum* (a) and *Hypnum plumaeforme* (b)

The research was conducted at the Heishiding Nature Reserve in Guangdong Province, southern China (23°27′ N, 111°53′ E), which has a monsoon climate (for details see Liu et al., 2015a). In December 2010, plants of the two selected moss species, *P. cirratum* (Sw.) Bird. subsp. *fuscatum* (Mitt.) Hyvönen (hereafter *P. cirratum*) and *H. plumaeforme* Wils. (Figure 1), were collected from locations near the experimental site, located in a valley bottom at the edge of the forest (Liu et al., 2011), and planted in 84 trays (30 cm × 60 cm, 42 trays per species). The mosses were then subjected to nitrogen and water deficit treatments as previously described (Liu et al., 2015a). Briefly, the mosses were acclimated at the experimental site for 2 months, during which they were exposed to ambient N deposition and watered every two days. Then, they were subjected to a monthly N supply treatment, in which nitrate (KNO_3_) or ammonium (NH_4_Cl) was applied to the trays at levels equivalent to 20, 40, or 60 kg N hm^−2^ year^−1^ (respectively, designated low, medium, and high +N treatments). Control sets (designated ‐N) received water only. Immediately after the final N or water application, sets of trays containing each species subjected to each N or control treatment were allocated to a water deficit or a control treatment, designated DS and −DS, respectively. After 12 days of these treatments, half of the moss samples in each tray were collected for physiological measurements; then, the remaining mosses in all the trays were watered with 200 ml distilled water every 2 days during a 10‐d recovery period, after which the rest of the samples were collected for measurements. The postrecovery −N/−DS, +N/−DS, −N/DS, and +N/DS samples were designated as R.−N/−DS, R.+N/−DS, R.−N/DS, and R.+N/DS, respectively.

### 2.2 Enzyme extraction

Enzyme extracts and chloroplast suspensions were prepared following methods described in the Handbook of the Shanghai Institute of Plant Physiology, CAS (SIPP, 1999). Briefly, about 1.5 g of fresh moss was cut from the top 2 cm of the individuals and ground in liquid nitrogen; then, 0.1 ml of 0.4 mol/l phenylmethanesulfonyl fluoride‐isopropanol solution and 8 ml of ice‐cold 50 mmol/l Tris–HCl (pH 7.5) buffer were added. After centrifuging the resulting suspension at 7000x g for 4 min at 4°C, the supernatant was used as the (soluble) enzyme extract, while a chloroplast suspension was prepared (for determining photophosphorylation activities) by resuspending the pellet in 4 ml of ice‐cold 50 mmol/l Tris–HCl (pH 7.5) buffer containing 2 mmol/l MgCl_2_, 10 mmol/l NaCl, 0.2 mol/l sorbitol, and 10% (*v/v*) glycerol.

### 2.3 Chlorophyll fluorescence and photophosphorylation activity measurements

Fresh moss samples were cut from the top 2 cm and used directly for chlorophyll fluorescence measurements. After adaptation in dark conditions for 20 min, the maximum fluorescence intensity (F_M_) and fluorescence intensities at 0 s (F_o_), 300 μs (F_300 μs_), and 2 ms (F_J_) were measured using a Plant Efficiency Analyzer (PEA; Hansatech Ltd., UK) with an excitation light intensity of 3000 mmol m^2^ s^1^ at ambient temperature. The performance index (on an absorption basis, expressed as PIabs) was calculated using Equation (1) and used for quantifying PS II behavior (Appenroth, Stöckel, Srivastava, & Strasser,^2001^; Strasser & Strasser, 1995).

(1) $$\mathrm{PIabs}={\left(F_{M}-F_{0}\right)}^{4}/\left[4F_{M}\;\cdot \;F_{0}\;\cdot \;\left(F_{300\;\mu s}-F_{0}\right)\cdot \left(F_{j}-F_{0}\right)\cdot \right]$$

CPSP (cyclic photophosphorylation) and NCPSP (noncyclic photophosphorylation) activities were calculated based on the consumption of inorganic phosphorus and represented by the synthesis of ATP (SIPP, 1999). Briefly, portions of chloroplast suspensions (prepared as described above) were added to Na_2_HPO_4_, ADP‐Na_2_ and 5‐methylphenazonium methosulfate (for CPSP measurements) or NADP‐Na and K_3_Fe(CN)_6_ (for NCPSP measurements). The inorganic phosphorus content in the reaction buffer was subsequently determined by phosphorus molybdenum blue colorimetry, using an ultraviolet spectrophotometer (WFZ UV‐2000, UNICO Ltd., Shanghai, China), which was also used for the following colorimetry measurements.

### 2.4 Carbon metabolism‐related enzymes

Ribulose‐1,5‐bisphosphate carboxylase (RuBPC, E.C. 4.1.1.39), glycolate oxidase (GO, E.C. 1.1.3.15), sucrose phosphate synthase (SPS, E.C. 2.4.1.14), and sucrose synthase (SS, E.C. 2.4.1.13) activities were measured according to SIPP (1999). To determine RuBPC activity, portions of enzyme extract (prepared as described above) were added to NaHCO_3_, NADH, ATP, creatine phosphate sodium, phosphocreatine kinase, 3‐phosphoglycerate kinase, and glyceraldehyde‐3‐phosphate dehydrogenase. The reaction was then initiated by adding RuBP, and the absorbance of the mixture at 340 nm was measured spectrophotometrically every minute for 3 min. The enzyme activity was calculated based on the consumption of NADH per min (SIPP, 1999). GO activity was measured by adding enzyme extract to a solution containing flavin mononucleotide, sodium glycolate, and phenylhydrazine hydrochloride, determining the glyoxylate phenylhydrazone product by K_3_Fe(CN)_6_ colorimetry at 550 nm, and expressing the activity in terms of glyoxylic acid (GA) generation. Uridine diphosphate glucose (added as the disodium salt) and D‐fructose 6‐phosphate were used as substrates for SPS activity assays and uridine diphosphate glucose and fructose as substrate for SS activity assays. The SPS and SS enzyme activities were measured and expressed in terms of sucrose generation.

### 2.5 Nitrogen metabolism‐related indices

Glutamine synthetase (GS, E.C. 2.7.7.42) activity was measured using a modified version of the SIPP (1999) method, adding enzyme extract to a solution containing Glu‐Na, NH_4_Cl, and ATP, determining the inorganic phosphorus generated via ATP degradation by phosphorus molybdenum blue colorimetry, and expressing the enzyme activity in terms of ATP consumption per min.

Glutamate dehydrogenase (GDH, E.C. 1.4.1.2) activity was measured via a modified version of the colorimetric method published by Moyano, Cárdenas, and Muñoz‐Blanco (1995), using α‐ketoglutaric acid and NH_4_Cl as the substrates and expressing the activity in terms of NADPH consumption per min.

To determine the plants' total N contents, samples of about 0.2 g were dried at 70°C for 12 hr and then digested with 5 ml H_2_SO_4_ and 1 ml H_2_O_2_ at 375°C until 30 min after the solution became colorless. After cooling, 10 ml of distilled water was added to the digested solution and the pH was adjusted to 7.0 with 5 mol/l NaOH. The resulting solution was diluted to 50 ml and then used to measure total N. A further 0.3 g of each moss sample was ground with 20 ml of 80% ethanol and then incubated in a water bath at 80°C for 30 min. The mixture was centrifuged at 1740x g for 5 min. A 10 ml portion of the supernatant was added to 2 ml 20% trichloroacetic acid and then diluted with distilled water to a final volume of 50 ml and filtered. A 25‐ml portion of the filtrate was digested as above, and the pH was adjusted to 7.0 with 5 mol/l NaOH after cooling and then diluted with distilled water to a final volume of 50 ml. This solution was used for determining NPN (nonprotein nitrogen) (SIPP, 1999).

Total N and NPN were analyzed using salicylic acid spectrophotometry (HJ 536‐2009), and PN (protein nitrogen) content was calculated as the difference between total N and NPN.

Free amino acids (FAAs) were extracted using a chloroform‐methanol solution according to Pérez‐Soba and de Visser (1994); then, total free amino acids (FAA), arginine (Arg), histidine (His), and proline (Pro) contents were, respectively, measured by ninhydrin colorimetry (Wang, 2006), naphthol colorimetry (He, Sun, & Chen, 2007), sulfanilamide colorimetry (Pan & Zhang, 2002), and acidic ninhydrin colorimetry, following Troll and Lindsley (1955) with minor modifications.

Details of the determination methods are provided in the Supporting Information.

### 2.6 Data analysis

All statistical analyses were carried out using SPSS 13.0 software. The relationships between N application and each physiological index associated with the −DS, DS, R.−DS, and R. DS water treatments were investigated using linear regression analysis. Differences in the indices associated between N treatment differences under each watering treatment were analyzed using one‐way ANOVA, and the LSD test was used to identify significant differences at the 0.05 probability level. Student's *t*‐test was used to detect significant differences (at 0.05 confidence limits) in physiological indices between watering treatments (−DS, DS, R.−DS, and R. DS) under the same N treatments.

## 3 Results

### 3.1 Chlorophyll a fluorescence transient

The PIabs of the mosses decreased with increasing N addition (Regression, *p *<* *.01, Table 1), the maximum reduction being 74% and 21% for ammonium‐ and nitrate‐treated *p. cirratum*, and 61% and 19% for ammonium‐ and nitrate‐treated *H. plumaeforme*, respectively. Water deficit stress also caused significant reductions in the mosses' PIabs (*t*‐test, *p *<* *.01).

**Table 1:** The performance index (PIabs) and activities of noncyclic photophosphorylation (NCPSP) and cyclic photophosphorylation (CPSP) in *Pogonatum cirratum* subsp. *fuscatum* and *Hypnum plumaeforme* exposed to indicated N treatments with or without water deficit stress (DS and −DS, respectively) and after a 10‐day recovery period from the treatments (R. DS and R.−DS, respectively). Data are presented as “mean ± S.D.” (*n* = 3), different letters indicate significant differences between N concentrations (*p *<* *.05, one‐way ANOVA, LSD test). Bold values in DS and R. DS groups represent significant differences between DS and −DS, R. DS and R.−DS, respectively (*p *<* *.05, *t*‐test)

N treatment (kg N hm^−2^ year^−1^)		Nitrate treatment			Ammonium treatment		
		PIabs	NCPSP (μmol ATP mg^−1 ^chl min^−1^)	CPSP (μmol ATP mg^−1 ^chl min^−1^)	PIabs	NCPSP (μmol ATP mg^−1 ^chl min^−1^)	CPSP (μmol ATP mg^−1 ^chl min^−1^)
*Pogonatum cirratum* subsp. *fuscatum*							
−DS	0	0.788 ± 0.052 b	0.26 ± 0.02 b	0.09 ± 0.00 b	0.788 ± 0.052 d	0.26 ± 0.02 c	0.09 ± 0.00 c
	20	0.753 ± 0.012 b	0.27 ± 0.01 b	0.07 ± 0.01 a	0.556 ± 0.036 c	0.20 ± 0.01 b	0.08 ± 0.01 c
	40	0.657 ± 0.021 a	0.25 ± 0.01 b	0.06 ± 0.02 a	0.360 ± 0.011 b	0.19 ± 0.01 ab	0.06 ± 0.01 b
	60	0.623 ± 0.047 a	0.22 ± 0.01 a	0.06 ± 0.01 a	0.202 ± 0.012 a	0.16 ± 0.02 a	0.03 ± 0.01 a
DS	0	**0.484 ± 0.031 c**	**0.17 ± 0.02 b**	**0.05 ± 0.01 c**	**0.484 ± 0.031 c**	**0.17 ± 0.02 b**	**0.05 ± 0.01 b**
	20	**0.437 ± 0.021 b**	**0.13 ± 0.02 a**	0.05 ± 0.02 c	**0.443 ± 0.032 c**	**0.12 ± 0.01 a**	**0.06 ± 0.01 b**
	40	**0.386 ± 0.021 b**	**0.12 ± 0.01 a**	**0.03 ± 0.00 b**	**0.233 ± 0.011 b**	**0.13 ± 0.01 a**	**0.03 ± 0.01 a**
	60	**0.342 ± 0.013 a**	**0.10 ± 0.01 a**	**0.02 ± 0.01 a**	**0.156 ± 0.009 a**	0.10 ± 0.02 a	0.02 ± 0.01 a
R.−DS	0	0.783 ± 0.031 d	0.24 ± 0.02 b	0.08 ± 0.01 b	0.783 ± 0.031 c	0.24 ± 0.02 a	0.08 ± 0.01 b
	20	0.649 ± 0.027 c	0.25 ± 0.01 b	0.09 ± 0.01 b	0.648 ± 0.037 b	0.25 ± 0.01 b	0.11 ± 0.01 b
	40	0.554 ± 0.022 b	0.24 ± 0.01 b	0.08 ± 0.01 b	0.349 ± 0.021 a	0.21 ± 0.02 a	0.09 ± 0.01 b
	60	0.455 ± 0.015 a	0.20 ± 0.01 a	0.06 ± 0.01 a	0.306 ± 0.019 a	0.20 ± 0.01 a	0.06 ± 0.01 a
R. DS	0	**1.449 ± 0.213 c**	**0.28 ± 0.01 b**	0.10 ± 0.01 b	**1.449 ± 0.213 d**	**0.28 ± 0.01 c**	0.10 ± 0.01 b
	20	**1.158 ± 0.101 c**	**0.28 ± 0.01 b**	**0.13 ± 0.02 b**	**0.956 ± 0.065 c**	**0.29 ± 0.01 c**	0.11 ± 0.01 b
	40	**0.952 ± 0.062 b**	**0.29 ± 0.01 b**	0.11 ± 0.02 b	**0.851 ± 0.051 b**	**0.26 ± 0.01 b**	0.10 ± 0.01 b
	60	**0.763 ± 0.024 a**	**0.24 ± 0.01 a**	0.07 ± 0.01 a	**0.664 ± 0.053 a**	0.21 ± 0.01 a	0.07 ± 0.01 a
*Hypnum plumaeforme*							
−DS	0	0.759 ± 0.055 b	0.31 ± 0.01 a	0.24 ± 0.01 b	0.759 ± 0.055 c	0.31 ± 0.01 d	0.24 ± 0.01 b
	20	0.733 ± 0.025 b	0.29 ± 0.02 a	0.22 ± 0.01 ab	0.666 ± 0.047 c	0.28 ± 0.01 c	0.26 ± 0.01 b
	40	0.689 ± 0.035 a	0.28 ± 0.01 a	0.23 ± 0.01 b	0.473 ± 0.033 b	0.26 ± 0.01 b	0.21 ± 0.01 a
	60	0.618 ± 0.041 a	0.30 ± 0.02 a	0.21 ± 0.01 a	0.293 ± 0.034 a	0.23 ± 0.01 a	0.19 ± 0.01 a
DS	0	**0.496 ± 0.023 c**	**0.21 ± 0.01 b**	**0.13 ± 0.01 c**	**0.496 ± 0.023 c**	**0.21 ± 0.01 b**	**0.13 ± 0.01 c**
	20	**0.435 ± 0.021 c**	**0.21 ± 0.02 b**	**0.12 ± 0.01 b**	**0.376 ± 0.039 b**	**0.20 ± 0.01 b**	**0.11 ± 0.01 b**
	40	**0.361 ± 0.027 b**	**0.22 ± 0.03 b**	**0.10 ± 0.01 ab**	**0.264 ± 0.018 a**	**0.18 ± 0.01 b**	**0.09 ± 0.01 ab**
	60	**0.288 ± 0.013 a**	**0.17 ± 0.01 a**	**0.09 ± 0.01 a**	**0.225 ± 0.028 a**	**0.15 ± 0.01 a**	**0.09 ± 0.01 a**
R.−DS	0	0.767 ± 0.061 a	0.31 ± 0.02 c	0.24 ± 0.01 c	0.767 ± 0.061 b	0.31 ± 0.02 d	0.24 ± 0.01 c
	20	0.761 ± 0.073 a	0.32 ± 0.02 c	0.22 ± 0.01 b	0.698 ± 0.057 b	0.29 ± 0.02 c	0.22 ± 0.01 b
	40	0.696 ± 0.043 a	0.28 ± 0.01 b	0.21 ± 0.01 b	0.566 ± 0.033 a	0.26 ± 0.01 b	0.20 ± 0.01 ab
	60	0.646 ± 0.053 a	0.25 ± 0.03 a	0.18 ± 0.01 a	0.533 ± 0.045 a	0.24 ± 0.01 a	0.19 ± 0.02 a
R. DS	0	**1.593 ± 0.213 c**	**0.50 ± 0.01 a**	**0.27 ± 0.01 b**	**1.593 ± 0.213 b**	**0.50 ± 0.01 b**	0.27 ± 0.01 b
	20	**1.268 ± 0.082 b**	**0.48 ± 0.02 a**	0.24 ± 0.01 b	**1.107 ± 0.124 a**	**0.48 ± 0.02 b**	0.24 ± 0.01 b
	40	**1.033 ± 0.104 a**	**0.48 ± 0.03 a**	0.21 ± 0.01 a	**0.924 ± 0.079 a**	**0.46 ± 0.02 a**	0.20 ± 0.01 a
	60	**0.936 ± 0.062 a**	**0.48 ± 0.01 a**	0.20 ± 0.01 a	**0.805 ± 0.085 a**	**0.45 ± 0.02 a**	0.19 ± 0.01 a

Increases in N supply also induced reductions in noncyclic photophosphorylation (NCPSP) and cyclic photophosphorylation (CPSP) activities in *P. cirratum* (Regression, *p *<* *.005, Table 1), but ammonium was associated with much larger declines than nitrate. In addition, NCPSP was inhibited by ammonium treatments (Regression, *p *<* *.001), but not nitrate addition, in *H. plumaeforme* samples. Water deficiency generally inhibited photophosphorylation activities of the mosses (*t*‐test, *p *<* *.05), but the inhibition was not significant in *P. cirratum* subjected to the low‐nitrate and high‐ammonium treatments (*t*‐test, *p *>* *.05), possibly due to relatively high standard deviations.

During recovery, PIabs increased in DS samples (*t*‐test, *p *<* *.001). In addition, PIabs was higher in R. DS samples than in corresponding R.−DS samples, by 85% and 107% in *P. cirratum* and *H. plumaeforme* samples, respectively, when no N was applied. However, N application decreased the differences between the R. DS and corresponding R.−DS samples.

NCPSP and CPSP activities in DS samples also increased during recovery (*t*‐test, *p *<* *.05), and NCPSP activities were higher in R. DS samples (except high ammonium‐treated *P. cirratum* samples) than in corresponding R.−DS samples (*t*‐test, *p *<* *.05, Table 1).

### 3.2 Photosynthetic carbon assimilation and photorespiration

**Figure 2:**
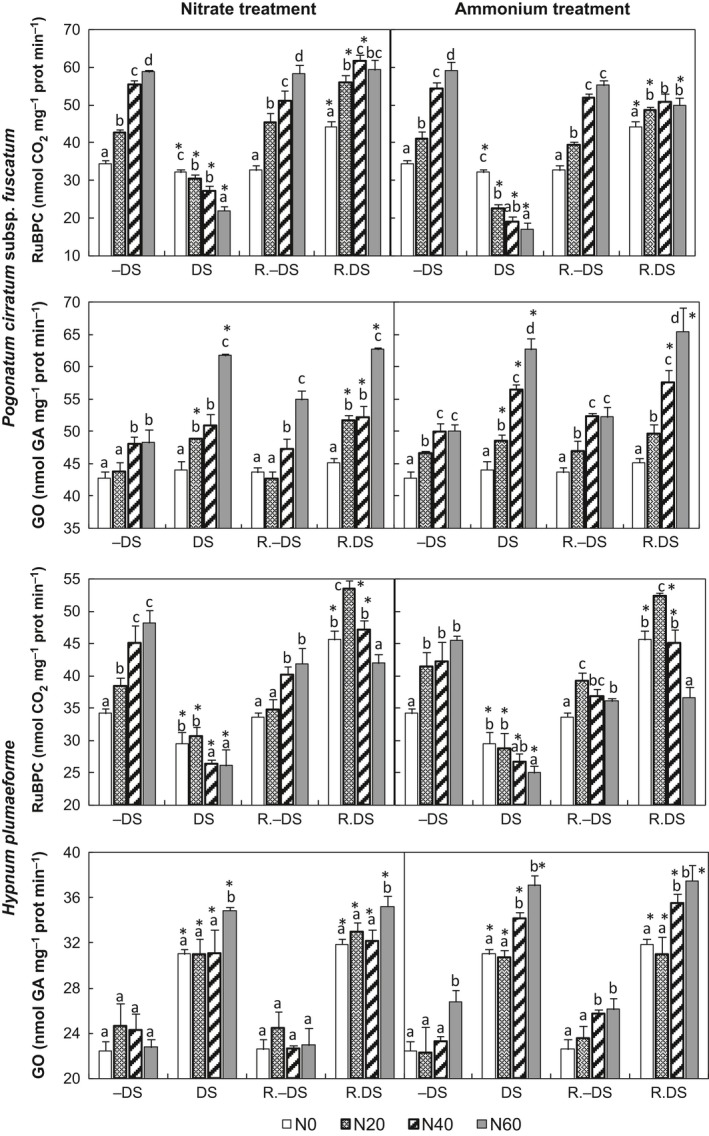
Activities of ribulose‐1,5‐bisphosphate carboxylase (RuBPC) and glycolate oxidase (GO) in *Pogonatum cirratum* subsp. *fuscatum* and *Hypnum plumaeforme* exposed to indicated N treatments with or without water deficit stress (DS and −DS, respectively) and after a 10‐day recovery period from the treatments (R. DS and R.−DS, respectively). N0, N20, N40, and N60 indicate N supply levels of 0, 20, 40, and 60 kg N hm^−2^ year^−1^, respectively. Data presented as “means + S.D.” (*n* = 3). Different letters above the bars indicate significant differences between N concentrations (*p *<* *.05, one‐way ANOVA, LSD test). Asterisks (*) above the DS and R. DS bars indicate significant differences between DS and −DS, R. DS and R.−DS, respectively (*p *<* *.05, *t*‐test)

RuBPC activity increased with increasing N supply in both *P. cirratum* and *H. plumaeforme* in the absence of water stress (Regression, *p *<* *.001). However, water deficit inhibited RuBPC activity (*t*‐test, *p *<* *.05), and combination with N supply caused a further decrease (Figure 2).

Application of both nitrate and ammonium increased GO activity in *P. cirratum* (Regression, *p *<* *.001), by up to 13% and 17%, respectively, in −DS conditions. The high ammonium treatment also caused an increase of 19% in GO activity in *H. plumaeforme* (ANOVA, *p *<* *.05; Figure 2). Water deficit caused a distinct increase in GO activities in *H. plumaeforme* (*t*‐test, *p *<* *.001) too, but it only increased GO activity in *P. cirratum* when additional N was supplied (*t*‐test, *p *<* *.01). Under DS conditions, N supply also stimulated GO activity in both species (Regression, *p *<* *.05).

During recovery, RuBPC activity in DS samples rose markedly (*t*‐test, *p *<* *.001), and it was much higher following recovery than in the corresponding R.−DS samples under no‐, low‐, or medium‐nitrogen additions (*t*‐test, *p *<* *.001 and *p *=* *.01, respectively), but the differences decreased with increase in N supply (Figure 2). The GO activity of DS samples did not differ significantly before and after recovery, but the GO activity in R. DS *H. plumaeforme* samples was much higher than in the corresponding R.−DS samples (*t*‐test, *p *<* *.001), and the activity in R. DS *P. cirratum* samples was also higher than in the corresponding R.−DS samples with added N (*t*‐test, *p *<* *.001; Figure 2).

### 3.3 Sucrose synthesis and degradation

**Figure 3:**
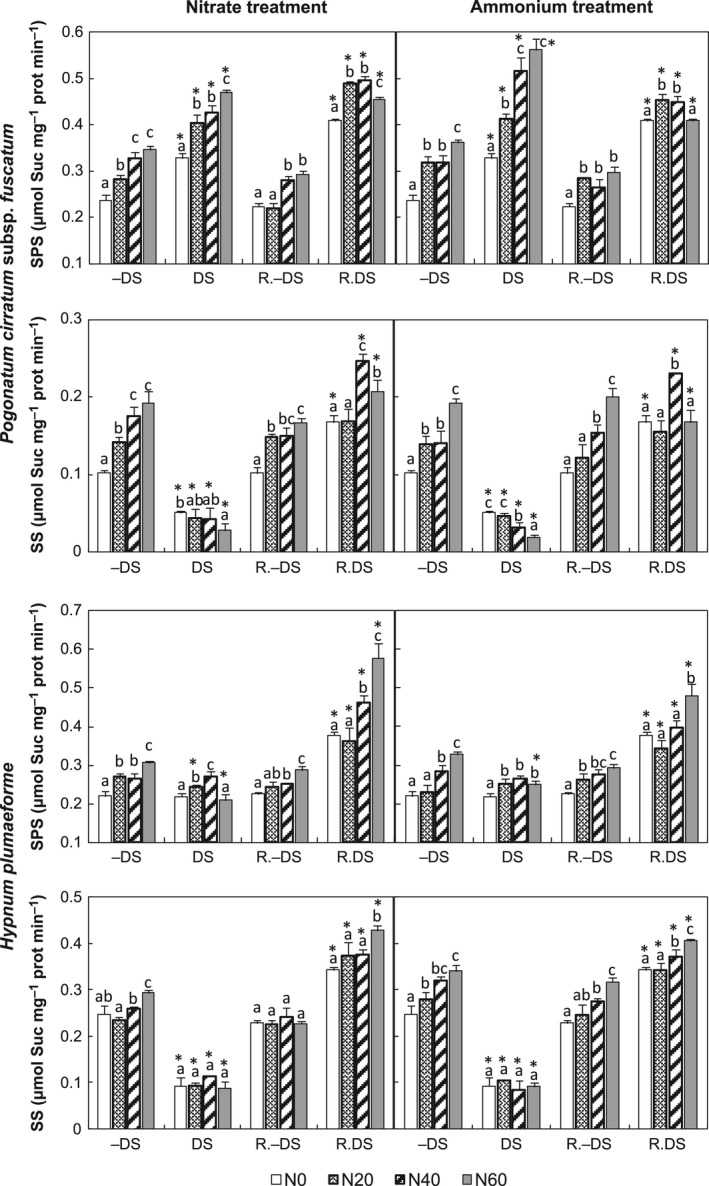
Activities of sucrose phosphate synthase (SPS) and sucrose synthase (SS) in *Pogonatum cirratum* subsp. *fuscatum* and *Hypnum plumaeforme* exposed to indicated N treatments with or without water deficit stress (DS and −DS, respectively) and after a 10‐day recovery period from the treatments (R. DS and R.−DS, respectively). N0, N20, N40, and N60 indicate N supply levels of 0, 20, 40, and 60 kg N hm^−2^ year^−1^, respectively. Data presented as “mean + S.D.” (*n* = 3). Different letters above the bars indicate significant differences between N treatment concentrations (*p *<* *.05, one‐way ANOVA, LSD test). Asterisks (*) above the DS and R. DS bars indicate significant differences between DS and −DS, R. DS and R.−DS, respectively (*p *<* *.05, *t*‐test)

N application stimulated SPS and SS activities in both species in the absence of water stress (Regression, *p *<* *.005), but it induced weaker increases in SPS activity (up to 52%) than in SS activity (up to 87%) in *P. cirratum*,, while in *H. plumaeforme* N supply stimulated SPS more than SS activity (up to 49% and 37%, respectively). Under water deficit conditions, supply of both N forms increased SPS activity and inhibited SS activity in *P. cirratum* (Regression, *p *<* *.05), but in *H. plumaeforme,* only ammonium significantly increased SPS activity (Regression, *p *<* *.05). SS activity of both species was strongly inhibited by water deficit, especially with added N (*t*‐test, *p *<* *.001). However, water deficit promoted SPS activity in *P. cirratum* (*t*‐test, *p *<* *.005), but slightly inhibited SPS activity in *H*. *plumaeforme* exposed to low nitrate or high N applications (*t*‐test, *p *<* *.05; Figure 3).

During short‐term recovery, SPS activity in DS *H. plumaeforme* samples strongly increased (*t*‐test, *p *<* *.001); however, in DS *P. cirratum* samples, it only increased significantly under non‐nitrogen, low‐nitrate, and medium‐nitrate treatments (*t*‐test, *p *<* *.01) and decreased under both medium‐ and high‐ammonium treatments (*t*‐test, *p *<* *.05). SS activity rose markedly during recovery in both species (*t*‐test, *p *<* *.05). Generally, SPS and SS activities of R. DS samples were higher than those of corresponding R.−DS samples (*t*‐test, *p *<* *.05, Figure 3).

### 3.4 Assimilation of inorganic nitrogen

**Figure 4:**
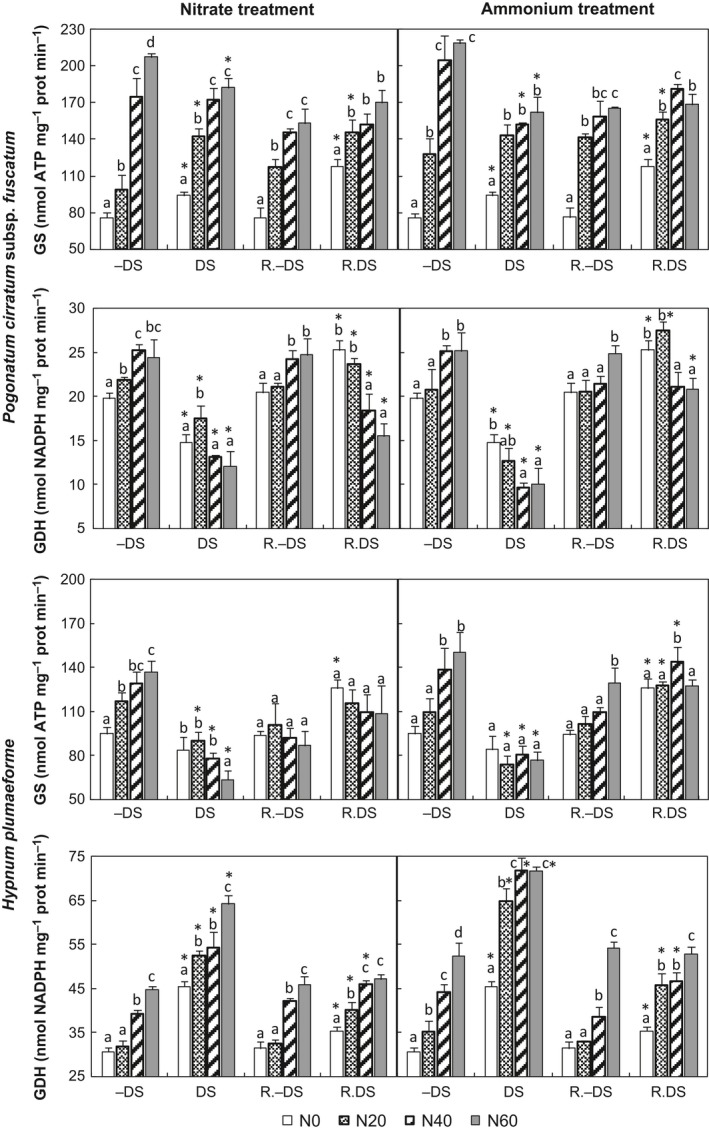
Activities of glutamine synthase (GS) and glutamate dehydrogenase (GDH) in *Pogonatum cirratum* subsp. *fuscatum* and *Hypnum plumaeforme* exposed to indicated N treatments with or without water deficit stress (DS and −DS, respectively) and after a 10‐day recovery period from the treatments (R. DS and R.−DS, respectively). N0, N20, N40, and N60 indicate N supply levels of 0, 20, 40, and 60 kg N hm^−2^ year^−1^, respectively. Data presented as “mean + S.D.” (*n* = 3). Different letters above the bars indicate significant differences between N concentrations (*p *<* *.05, one‐way ANOVA, LSD test). Asterisks (*) above the DS and R. DS bars indicate significant differences between DS and −DS, R. DS and R.−DS, respectively (*p *<* *.05, *t*‐test)

As shown in Figure 4, N application without water stress caused clear increases in the GS activity of both species (Regression, *p *<* *.001), but far higher increases in *P. cirratum* than in *H. plumaeforme*. GS activity also increased (but much less strongly) with increase in N supply in DS *P. cirratum* samples (Regression, *p *<* *.001). In *P. cirratum*, GS activity was higher under non‐nitrogen or low‐nitrate treatments, and lower under high‐nitrate or medium‐ammonium and high‐ammonium treatments in DS samples than in the corresponding –DS samples (*t*‐test, *p *<* *.01). In *H. plumaeforme*, N application did not stimulate GS activity in DS samples (Regression, *p *>* *.05) and it was clearly lower than the corresponding –DS samples (*t*‐test, *p *<* *.001).

N applications induced increases in the GDH activity of both species (Regression, *p *=* *.001), but higher increases in *H. plumaeforme* than in *P. cirratum*. Water deficit promoted GDH activity in *H. plumaeforme*, but inhibited GDH activity in *P. cirratum* (*t*‐test, *p *<* *.001), and the effects were more pronounced under ammonium treatment. GDH activity increased with increase in N supply in DS samples of *H. plumaeforme* (Regression, *p *<* *.001). Under DS conditions, low nitrate supply stimulated GDH activity slightly and medium‐ or high‐ammonium treatments significantly inhibited GDH activity in *P. cirratum* samples (ANOVA, *p *<* *.05, Figure 4).

During short‐term recovery, GS activity did not change significantly in DS samples of *P. cirratum* (*t*‐test, *p *>* *.05), but it increased in DS samples of *H. plumaeforme* (*t*‐test, *p *<* *.01). GS activities in R. DS samples were higher than (or similar to) those of the corresponding R.−DS samples (Figure 4).

During recovery, GDH activity clearly increased in DS samples of *P. cirratum* (*t*‐test, *p *<* *.01), but clearly declined in those of *H. plumaeforme* (*t*‐test, *p *<* *.001). GDH activity was slightly higher under non‐ or low‐N treatments**,** and similar or lower under medium‐ or high‐nitrogen treatments in R. DS samples of *P. cirratum* than in corresponding R.−DS samples. However, GDH activity was higher in R. DS samples of *H. plumaeforme* than in the corresponding R.−DS samples generally (*t*‐test, *p *<* *.05, except for high N‐treated samples) (Figure 4).

### 3.5 Tissue nitrogen contents

NPN content increased with increase in N supply (Regression, *p *<* *.001; Table 2), but PN content remained stable or declined; thus, the NPN/PN ratio increased with increase in N supply in both −DS and DS samples (Regression, *p *<* *.001).

**Table 2:** Contents of protein‐N (PN) and nonprotein‐N (NPN) in *Pogonatum cirratum* subsp. *fuscatum* and *Hypnum plumaeforme* exposed to indicated N treatments with or without water deficit stress (DS and −DS, respectively) and after a 10‐day recovery period from the treatments (R. DS and R.−DS, respectively). Data are presented as “mean ± S.D.” (*n* = 3), different letters indicate significant differences between N concentrations (*p *<* *0.05, one‐way ANOVA, LSD test). Bold values in DS and R. DS groups represent significant differences between DS and −DS, R. DS and R.−DS, respectively (*p *<* *0.05, *t*‐test)

N treatment (kg N hm^−2^ year^−1^)		Nitrate treatment		Ammonium treatment	
		PN (mmol g^−1 ^Fw)	NPN (μmol g^−1 ^Fw)	PN (mmol g^−1 ^Fw)	NPN (μmol g^−1 ^Fw)
*Pogonatum cirratum* subsp. *fuscatum*					
−DS	0	0.101 ± 0.007 a	19.73 ± 1.31 a	0.101 ± 0.007 a	19.73 ± 1.31 a
	20	0.093 ± 0.008 a	23.58 ± 0.63 b	0.092 ± 0.007 a	31.64 ± 2.06 b
	40	0.103 ± 0.002 a	26.58 ± 1.27 c	0.089 ± 0.003 a	42.23 ± 1.93 c
	60	0.102 ± 0.005 a	28.99 ± 1.22 c	0.090 ± 0.003 a	45.04 ± 3.51 c
DS	0	**0.084 ± 0.004 a**	20.24 ± 1.94 a	**0.084 ± 0.004 a**	20.24 ± 1.94 a
	20	0.085 ± 0.003 a	25.68 ± 1.70 b	**0.072 ± 0.005 a**	32.33 ± 1.68 b
	40	**0.085 ± 0.002 a**	**29.47 ± 0.77 c**	**0.077 ± 0.001 a**	**37.52 ± 0.98 c**
	60	**0.083 ± 0.005 a**	**35.14 ± 1.09 d**	0.075 ± 0.012 a	41.21 ± 1.72 d
R.−DS	0	0.097 ± 0.005 a	18.50 ± 2.90 a	0.097 ± 0.01 a	18.50 ± 1.90 a
	20	0.095 ± 0.009 a	17.88 ± 1.87 a	0.090 ± 0.02 a	21.96 ± 0.72 a
	40	0.089 ± 0.009 a	16.54 ± 0.59 a	0.083 ± 0.01 a	28.89 ± 2.07 b
	60	0.096 ± 0.008 a	23.98 ± 0.91 b	0.082 ± 0.01 a	32.23 ± 0.82 c
R. DS	0	0.094 ± 0.005 a	18.61 ± 0.88 a	0.094 ± 0.005 b	18.61 ± 0.88 a
	20	0.100 ± 0.014 a	19.60 ± 1.31 a	0.085 ± 0.015 ab	20.47 ± 0.88 a
	40	0.100 ± 0.005 a	**20.92 ± 1.34 ab**	0.085 ± 0.011 ab	26.28 ± 0.68 b
	60	0.100 ± 0.001 a	23.07 ± 1.00 b	**0.072 ± 0.009 a**	**43.50 ± 2.94 c**
*Hypnum plumaeforme*					
−DS	0	0.097 ± 0.005 b	10.35 ± 0.62 a	0.097 ± 0.005 a	10.35 ± 0.62 a
	20	0.081 ± 0.006 a	23.23 ± 1.66 b	0.097 ± 0.003 a	19.01 ± 2.27 b
	40	0.074 ± 0.010 a	36.52 ± 2.85 c	0.095 ± 0.001 a	33.56 ± 1.54 c
	60	0.074 ± 0.005 a	40.94 ± 0.95 d	0.098 ± 0.005 a	46.74 ± 2.07 d
DS	0	0.106 ± 0.006 b	**25.14 ± 1.13 a**	0.106 ± 0.006 c	**25.14 ± 1.13 a**
	20	**0.060 ± 0.003 b**	**34.79 ± 1.49 b**	**0.067 ± 0.005 b**	**38.29 ± 2.75 b**
	40	0.058 ± 0.010 ab	**50.17 ± 1.94 c**	**0.054 ± 0.012 ab**	**47.16 ± 0.96 c**
	60	**0.051 ± 0.005 a**	**50.22 ± 0.51 c**	**0.047 ± 0.008 a**	**59.33 ± 1.27 d**
R.−DS	0	0.093 ± 0.011 b	10.59 ± 0.28 a	0.093 ± 0.011 a	10.59 ± 0.28 a
	20	0.071 ± 0.008 a	20.01 ± 1.87 b	0.094 ± 0.008 a	21.35 ± 1.75 b
	40	0.063 ± 0.009 a	24.76 ± 1.02 c	0.087 ± 0.010 a	28.42 ± 0.96 c
	60	0.054 ± 0.005 a	36.10 ± 2.39 d	0.084 ± 0.007 a	32.75 ± 1.54 d
R. DS	0	**0.112 ± 0.012 a**	**24.63 ± 1.69 a**	**0.112 ± 0.012 a**	**24.63 ± 1.69 a**
	20	**0.123 ± 0.007 a**	**32.81 ± 1.68 b**	**0.149 ± 0.006 b**	**40.93 ± 5.01 b**
	40	**0.127 ± 0.004 a**	**31.16 ± 1.59 b**	**0.170 ± 0.009 c**	**41.50 ± 1.59 c**
	60	**0.140 ± 0.005 b**	40.46 ± 2.83 c	**0.161 ± 0.005 bc**	**49.25 ± 1.15 d**

DS treatment generally decreased the PN content but stimulated accumulation of NPN in the mosses (*t*‐test, *p *<* *.05; Table 2).

During the short recovery period, the NPN content in N‐treated DS samples generally declined (*t*‐test, *p *<* *.001), while the PN content in DS *H. plumaeforme* samples increased significantly (*t*‐test, *p *<* *.001), and the increase in PN content was larger under ammonium treatments. NPN and PN contents in R. DS *P. cirratum* samples were similar to those of the corresponding R.−DS samples (*t*‐test, *p *>* *.05, except for samples subjected to the medium‐nitrate and high‐ammonium treatments). However, NPN and PN contents were higher in R. DS *H. plumaeforme* samples than the corresponding R.−DS samples (*t*‐test, *p *<* *.001, except for those subjected to the high‐nitrate treatment, Table 2).

### 3.6 Amino acid contents

**Figure 5:**
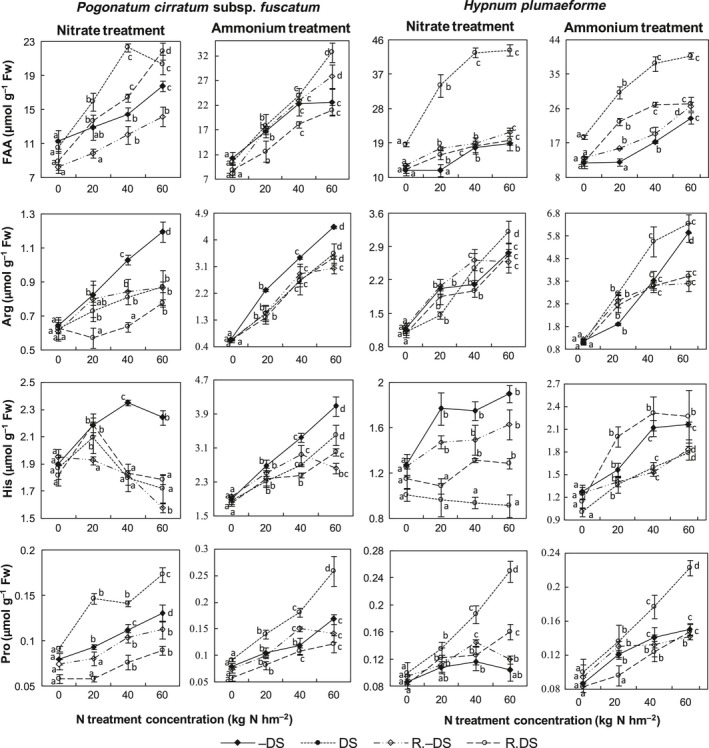
Contents of total free amino acids (FAA), arginine (Arg), histidine (His), and proline (Pro) in *Pogonatum cirratum* subsp. *fuscatum* and *Hypnum plumaeforme* exposed to indicated N treatments with or without water deficit stress (DS and −DS, respectively) and after a 10‐day recovery period from the treatments (R. DS and R.−DS, respectively). Data presented as “mean ± S.D.” (*n* = 3). For each water treatment, different letters indicate significant between‐concentration differences (*p *<* *.05, one‐way ANOVA, LSD test)

As shown in Figure 5, FAA contents in −DS samples of both species increased with increase in N supply (Regression, *p *<* *.001), by up to around 58% and 99% under nitrate and ammonium treatments, respectively. Arg, His, and Pro contents also increased with increase in N supply (Regression, *p *<* *.05), but much more strongly in ammonium‐treated samples than in nitrate‐treated samples. Total FAA, Arg, and Pro contents in DS samples also increased with increase in N supply (Regression, *p *<* *.001), but nitrate treatments had no effect on His content (Regression, *p *>* *.05). When no N was supplied, contents of the amino acids in DS and −DS samples did not differ significantly (*t*‐test, *p *>* *.05), but when N was applied, FAA and Pro contents were higher, while Arg and His contents were lower, in DS than in −DS samples of *P. cirratum* (*t*‐test, *p *<* *.05). *H. plumaeforme* showed similar trends in FAA, Pro, and His contents, but not Arg contents (Figure 5).

During the recovery period, FAA and Pro contents declined significantly (*t*‐test, *p *<* *.001), but Arg contents only declined in DS *P. cirratum* samples subjected to nitrate treatment (*t*‐test, *p *<* *.001). His content showed no significant changes in DS *P. cirratum* samples during recovery (*t*‐test, *p *>* *.05). In DS samples of *H. plumaeforme*, FAA content decreased and His content increased significantly, and in DS samples of *H. plumaeforme* subjected to ammonium, medium‐nitrate, and high‐nitrate treatments, Arg and Pro contents decreased significantly during recovery (*t*‐test, *p *<* *.001). Pro contents were generally lower in R. DS samples of *P. cirratum* than in corresponding R.−DS samples (*t*‐test, *p *<* *.01). However, no obvious patterns were observed in the differences in Pro contents between R. DS and corresponding R.−DS samples of *H. plumaeforme* (Figure 5).

## 4 Discussion

### 4.1 Photosystem II (PSII), photosynthetic carbon assimilation, and carbohydrate synthesis

N supply generally decreased the mosses' performance (PIabs) and rates of both cyclic photophosphorylation (CPSP) and noncyclic photophosphorylation (NCPSP), presumably due to oxidative stress caused by excess N (Liu et al., 2015a) damaging PSII. However, ammonium caused stronger declines than nitrate, possibly because photosynthetic electron transport and photophosphorylation can be uncoupled by excess NH_4_^+^ (Good, 1977), but not excess nitrate (Macnab, Lawlor, Baker, & Young, 1987). Nevertheless, N application stimulated the activity of ribulose‐1,5‐bisphosphate carboxylase (RuBPC), the first and rate‐limiting reaction of photosynthetic carbon assimilation. Furthermore, sucrose phosphate synthase (SPS) and sucrose synthase (SS) activities of the mosses increased under N treatments, indicating that N application accelerated the turnover rate of sucrose, as SPS is a key enzyme in the sucrose biosynthetic pathway, while SS mainly participates in sucrose decomposition (Touchette & Burkholder, 2007).

Water deficit stress led to sharp decreases in PIabs, NCPSP, and CPSP in the mosses, possibly due to rupturing of thylakoid membranes (Ladjal, Epron, & Ducrey, 2000) and degradation of D1‐D2 dimers (Lu & Zhang, 1999). Water deficit stress also severely inhibited RuBPC activity, in accordance with previous studies (Majumdar, Ghosh, Glick, & Dumbroff, 1991; Parry, Andralojc, Khan, Lea, & Keys, 2002; Stitt & Krapp, 1999). In combination with N treatments, water deficit caused further reductions in PIabs, NCPSP, CPSP, and RuBPC, demonstrating that N application strengthened the adverse effects of water stress. This contrasts with findings by Iqbala, Umara, and Khan (2015) that excess N led to a recovery in RuBPC activity inhibited by salinity stress in mustard (*Brassica juncea*). Thus, we conclude that effects of nitrogen on Rubisco activity depend on complex interactions between N levels, environmental factors and plant species mediated by the extremely complex regulatory network that controls interactions between C and N metabolism.

Although water deficit treatment increased or had no significant effect on SPS activity, it distinctly inhibited SS activity and the inhibition was strengthened when combined with N application. These findings indicate that water deficit may induce sucrose accumulation in the mosses, especially when N is readily available, which may help the plants to resist osmotic stress (Guy, Huber, & Huber, 1992).

During short‐term recovery from water deficit stress, PIabs, NCPSP, CPSP, and RuBPC in DS samples of both mosses increased and PIabs, NCPSP, and RuBPC in R. DS samples were higher than the corresponding R.−DS samples, indicating that there were overcompensation effects in recovery of the PSII and C assimilation machinery. However, N application strongly decreased the degree of compensation of both PSII and C assimilation functionality after recovery. Meanwhile, SPS overcompensation effects were observed following recovery from water deficit stress under all N treatment conditions. As sucrose synthesis catalyzed by SPS plays an important role in rapid plant growth (Touchette & Burkholder, 2007), a compensatory increase in SPS activity probably makes important contributions to compensatory growth of the mosses after stress.

Glycolate oxidase (GO) is one of the most important enzymes involved in photorespiration. N application and water deficit increased GO activity both singly and synergistically, indicating that photorespiration was an important mechanism preventing photic damage to PSII under the stress conditions we imposed. GO activity remained relatively high after short‐term recovery from water deficit, suggesting that photorespiration continued to provide important protection for PSII during the recovery period.

### 4.2 Inorganic nitrogen assimilation and amino acid transformation

Glutamine synthetase (GS), glutamate synthase (GOGAT) (Fig. S1), and glutamate dehydrogenase (GDH) activities in the mosses all increased significantly with increase in N supply, in accordance with previous findings (Touchette & Burkholder, 2007). N application also distinctly increased NPN contents, but had no effect on PN, indicating that the assimilated N was mainly stored as free amino acids, especially amino acids with a high N/C ratio, such as Arg and His, which play important roles in nitrogen storage and transport under excess N conditions (Koranda et al., 2007; Paulissen et al., 2005).

Our results showed that water stress inhibited activity of the GS/GOGAT pathway and stimulated GDH activity in *H. plumaeforme*, and combining N addition with water stress strengthened these responses. However, *P. cirratum* displayed almost opposite patterns. As the GDH pathway consumes significantly less energy than the GS/GOGAT pathway (Helling, 1994), our results suggest that when stressed, *H. plumaeforme* preferentially uses a low–energy‐consuming N assimilation pathway, while *P. cirratum* preferentially uses a high‐energy‐consuming pathway. *Pogonatum cirratum* and *H. plumaeforme* also preferentially synthesize antioxidative secondary metabolites that require high‐ and low‐energy inputs, respectively, when subjected to oxidative stress (Liu et al., 2015a,b). Thus, differences in the energy utilization strategies adopted by the two species may explain their difference in selection of N assimilation pathway under stressed conditions. However, more detailed investigations are needed to elucidate the mechanisms involved.

Photorespiration generates significant quantities of NH_4_^+^ in the conversion of glycine to serine, and the released NH_4_^+^ is refixed via the same pathway as the primary N assimilation (Nunes‐Nesi, Fernie, & Stitt, 2010). These processes have been referred to as the photorespiratory N cycle (Keys, 2006; Restivo, 2004). We found that both water stress and N addition stimulated photorespiration, thereby complicating regulation of N assimilation, in the studied mosses.

We also found that water stress decreased PN contents generally, but increased NPN and FAA contents of the mosses, especially under low‐to‐moderate N supply conditions, indicating that endogenous proteolysis activity was stimulated by oxidative stress (Palma et al., 2002). This would clearly induce accumulation of FAA, one of the drought‐resistance mechanisms employed by mosses (Rai, 2002; Ramanjulu & Sudhakar, 1997; Xu, Zhou, Liu, & Chen, 2011; Yoshiba, Kiyosue, Nakashima, Yamaguchi‐Shinozaki, & Shinozaki, 1997), and readily available N may enhance this resistance pathway.

In addition, proline (Pro) participates in plants' osmotic adjustment (Mittal, Kumari, & Sharma, 2012) and plays an important role in their antioxidation mechanisms (Chen & Dickman, 2005). Both N application and water deficit caused an increase in Pro content, and the differences in Pro content between DS and −DS samples increased with increase in N supply, in accordance with previous findings regarding oxidative stress (Liu et al., 2015a).

After recovery from the water stress, the inhibited nitrogen assimilation pathways in the mosses exhibited an apparent compensation effect. However, N application significantly decreased the compensatory degree of N assimilation. On the other hand, during the recovery from water stress, PN content increased but FAA contents in both mosses decreased significantly while the NPN/PN ratio decreased in *H. plumaeforme*, indicating that protein synthesis had begun to recover (Good & Zaplachinski, 1994). In addition, Pro content decreased after recovery, indicating a reduction in the degree of oxidative stress.

## 5 Conclusion

N application stimulated carbon and nitrogen assimilation, although excess N damaged the PSII reactive centers of the mosses. Water deficit had significant negative effects on the studied mosses**,** inducing damage to their PSII reaction centers and adversely affecting their C and N assimilation, although an interspecific difference in N assimilation pathway preference was found. However, our results also show that the mosses have several mechanisms that enable them to cope with the stress, including stimulation of photorespiration and increasing Pro content. In addition, the mosses displayed a certain level of resilience to water deficit stress, and overcompensation effects were generally found after rehydration.

However, the combined stress of water deficit and excess N exacerbated the negative effects on the mosses, causing further decreases in C and N assimilation and further increases in photorespiration rates, thus seriously reducing the mosses' growth. Furthermore, after rehydration, the recovery capacity of the mosses subjected to combined N treatments decreased. These results suggest that increases in N deposition may amplify the damages caused by water deficit in the mosses, and when evaluating effects of changes in rainfall patterns on mosses, N deposition should also be cautiously considered.

## Conflict of Interest

None declared.
