# Supplementary material for: Physiological responses of two moss species to the combined stress of water deficit and elevated N deposition (II): Carbon and nitrogen metabolism
Source: Ecol Evol. 2016 Oct 4;6(21):7596–609. doi: 10.1002/ece3.2521 (PMC6093146; doi:10.1002/ece3.2521)
Supplement: Supplementary file 1 [file ECE3-6-7596-s001.pdf]

Supporting information 1: Details of the methods for determining physiological indices

Photophosphorylation activity

CPSP (cyclic photophosphorylation) and NCPSP (noncyclic photophosphorylation) activity was calculated on the basis of consumption of inorganic phosphorus, and represented by the synthesis of ATP (SIPP, 1999). One ml of the chloroplast suspension was added into 1 ml of 0.05 M Tris-HCl buffer (pH 7.5) containing 1 mM $MgCl_2$, 0.5 mM Na_2HPO_4, 0.5 mM ADP-Na_2, 0.03 mM 5-Methylphenazonium methosulfate (for CPSP) /0.5 mM NADP-Na and 0.5 mM $K_3Fe(CN)_6$ (for NCPSP). After 30 min of illumination (60 μmol m^{-2} s^{-1}, at room temperature), 1 ml of 20% trichloroacetic acid was added to halt the reaction. The reaction buffer was centrifuged at 12000 rpm for 2 min, and 2.5 ml of the coloration buffer (containing 6% $FeSO_4$, 3% $(NH_4)_6Mo_7O_{24}$ and 1.5 M H_2SO_4) was added to 0.5 ml of the supernatant, then the absorbance was quantified spectrophotometrically at 660 nm to determine the inorganic phosphorus content.

Mg^{2+}-ATPase (MAE, E.C. 3.6.1.3) activity was measured according to the method described in the handbook of the Shanghai Institute of Plant Physiology, CAS (SIPP, 1999). A portion of 0.5 ml chloroplast suspension was added to 1 ml of reaction buffer containing 0.05 M Tris-HCl (pH 8.0), 3 mM $MgCl_2$, 10 mM NaCl, 3 mM ATP and 0.2% (v/v) methanol, after incubation at 30 ºC for 30 min, 1 ml 20% trichloroacetic acid was added and then the inorganic phosphorus content was measured as above. MAE activity was calculated by the generation of inorganic phosphorus in 30 min.

Activities of Carbon metabolism related enzymes

To determine the activity of Ribulose-1,5-bisphosphate carboxylase (RuBPC, E.C. 4.1.1.39), 80 μl enzyme extract was added to 0.5 ml 50 mM Tris-HCl buffer (pH 8.2) containing 10 mM $MgCl_2$, 0.6 mM dithiothreitol, 14 mM $NaHCO_3$ and 0.4 mM NADH, after equilibration at 25 ºC for 5 min, solutions of 70 μl 6 mM ATP, 70 μl 6 mM creatine phosphate sodium, 35 μl 160 U ml^{-1} phosphocreatine kinase, 35 μl 160 U ml^{-1} 3-phosphoglycerate kinase, and 35 μl 160 U ml^{-1} glyceraldehyde-3-phosphate dehydrogenase were added successively. The reaction was finally initiated by adding 35 μl 9 mM RuBP and then the absorbance of the mixture was measured spectrophotometrically at 340 nm every minute for 3 minutes. The enzyme activity was calculated based on the NADH consumption per min (SIPP, 1999).

Glycolate oxidase (GO, E.C. 1.1.3.15) activity was measured according to SIPP (1999). A portion of 0.4 ml enzyme extract was added to 0.7 ml 0.1 M PBS (pH 8.0) buffer, and then 0.1 ml 0.7 mM flavin mononucleotide and 0.1 ml 35 mM phenylhydrazine hydrochloride were added. After equilibration at 30ºC for 10 min, 0.1 ml 35 mM sodium glycollate was added to initiate the reaction. After 10 min of reaction, 0.1 ml of 2 M HCl was added to halt the reaction, and 1 ml of concentrated hydrochloric acid and 0.2 ml of 0.05 M $K_3Fe(CN)_6$ were added to the buffer, then the absorbance at 550 nm was measured spectrophotometrically after 20 min.

Sucrose phosphate synthase (SPS, E.C. 2.4.1.14) activity was measured according to SIPP (1999). A portion of 0.1 ml enzyme extract was added to 0.15 ml 50 mM Tris-HCl buffer (pH 7.0) containing 10 mM $MgCl_2$, 7 mM uridine diphosphate glucose and 10 mM D-fructose 6-phosphate disodium salt, and was incubated at 30 ºC for 30 min, then mixed with 0.05 ml 2 M NaOH and boiled for 10 min. After cooling, the mixture was added to 0.7 ml 30% HCl and 0.2 ml 0.1% resorcinol-alcohol solution, and incubated at 80 ºC for 10 min, then analysed spectrophometrically at 480 nm after cooling. The activity was represented by the generation of sucrose.

Sucrose synthase (SS, E.C. 2.4.1.13) activity was measured according to SIPP (1999). The measurement process was same as for SPS except that the buffer contained 10 mM fructose instead of 10 mM D-fructose 6-phosphate disodium salt.

N metabolism related indices

Glutamine synthetase (GS, E.C. 2.7.7.42) activity was measured according to a modified version of the SIPP (1999) method. A portion of 0.6 ml 50 mM Tris-HCl buffer (pH 7.5) containing 0.25 M Glu-Na and 0.15 M NH_4Cl was mixed with 0.1 ml 0.1 M ATP, 0.1 ml 1 M $MgSO_4$ and 0.4 ml enzyme extract, and incubated at 37 ºC for 30 min, then 0.8 ml of 20% trichloroacetic acid was added to halt the reaction and the mixture was centrifuged at 10000 rpm for 5 min. One ml of the supernatant was mixed with 2.5 ml coloration buffer (containing 6% $FeSO_4$, 3% $(NH_4)_6Mo_7O_{24}$ and 1.5 M H_2SO_4), the inorganic phosphorus content was analysed spectrophotometrically at 660 nm. The control was treated with the same reagents and conditions except that the enzyme extract was added after the trichloroacetic acid. The enzyme activity was defined as the difference between the reaction and the control and represented as the ATP consumed per min.

Glutamate dehydrogenase (GDH, E.C. 1.4.1.2) was measured using a modified version of the Moyano et al. (1995) method. A portion of 0.86 ml 50 mM Tris-HCl reaction buffer (pH 8.2) containing 50 mM α-ketoglutaric acid and 40 mM NH_4Cl was mixed with 75 µl enzyme extract and incubated at 30 °C for 2 min. Then, 65µl 10 mM NADPH solution was added to initiate the reaction. Absorbance at 340 nm was measured spectrophotometrically immediately, and then measured once per minute for 3 minutes. The enzyme activity was defined as the NADPH consumed per min.

To determine total N in the plants, samples weighing about 0.2 g were dried at 70°C for 12 h and then digested with 5 ml H_2SO_4 and 1 ml H_2O_2 at 375°C until 30 min after the digested solution became colourless. After cooling, 10 ml of distilled water was added to the digested solution and the pH was adjusted to 7.0 with 5 M NaOH. The resulting solution was diluted to 50 ml and then used to measure total N. A further 0.3 g of moss was ground with 20 ml of 80% ethanol and then incubated in a water bath at 80 °C for 30 min. The mixture was subjected to centrifugation at 5000 rpm for 5 min. A 10 ml portion of the supernatant was added to 2 ml 20% trichloroacetic acid, and then diluted with distilled water to a final volume of 50 ml before being filtered. A portion of 25 ml filtrate was digested as above, the pH was adjusted to 7.0 with 5 M NaOH after cooling and then diluted with distilled water to a final volume of 50 ml. The solution was used for the determination of NPN (SIPP 1999).

Total N and NPN (non-protein nitrogen) were analysed using salicylic acid spectrophotometry. To a 2 ml portion of the total N (or NPN) extract, 2 ml of 0.01 M NaOH, 1 ml salicylic acid reagent (5% (*m/v*) salicylic acid and 5% (*m/v*) seignette salt), 0.1 ml 1.8% (*m/v*) sodium nitroprusside solution and 0.1 ml NaClO solution (containing 0.35% available chlorine) were successively added, and the resulting mixture was left to react for 2-3 h. The solution was analysed spectrophotometrically at 697 nm using ammonia as the standard (HJ 536-2009). PN (protein nitrogen) content was calculated as the difference between total N and non-protein N.

FAAs (free amino acids) were extracted according to Pérez-Soba & de Visser (1994). About 1.0 g of moss sample was ground with 30 ml extract solution (chloroform : methanol : distilled water = 37 : 48 : 15) and filtered. The filtrate was transferred to a separating funnel, to which was added 4.7 ml chloroform and 6 ml distilled water, and then left to stand at room temperature until it had delaminated. The aqueous phase was used to determine FAA contents.

Total FAA content was measured according to Wang (2006). A 0.4 ml portion of the extract was mixed

with 1 ml acetate buffer (pH 5.4, containing 0.08% $SnCl_2$) and 1 ml of 2% ninhydrin in ethanol, then boiled for 15 min. After cooling, the mixture was added to 7 ml of 0.2% KIO_3 solution (in 70% ethanol) and analysed spectrophotometrically at 630 nm, using leucine as a standard.

Arginine (Arg) content was measured according to He et al. (2007). Briefly, 2 ml distilled water, 1 ml 5% α-naphthol (dissolved in 4% NaOH) and 1 ml of 0.02% butanedione (containing 0.02% acetone) were successively added to a 2 ml aliquot of the extract. The mixture was incubated at 30°C for 30 min and then analysed spectrophotometrically at 525 nm .

Histidine (His) content was measured according to Pan & Zhang (2002). A 2 ml portion of the extract was added to 0.2 ml of 1% sulfanilamide and 0.2 ml of 5% $NaNO_2$ and reacted for 15 min. The mixture was added to 0.6 ml of 15% Na_2CO_3 and 2 ml of 20% ethanol, left to stand for 5 min and then analysed spectrophotometrically at 510 nm.

Proline (Pro) content was determined using a modified version of the Troll & Lindsley (1955) method. A portion (2 ml) of the extract was mixed with 2 ml glacial acetic acid and 2 ml of 2% ninhydrin in ethanol-glacial acetic- phosphoric acid solution (the volume ratio = 25 : 60 : 15) and boiled for 40 min. After cooling, 3.5 ml of toluene was added to the mixture, which was then shaken vigorously and left to stand until it had delaminated. The toluene phase was collected and added to 0.5 ml methanol and then analysed spectrophotometrically at 520 nm.

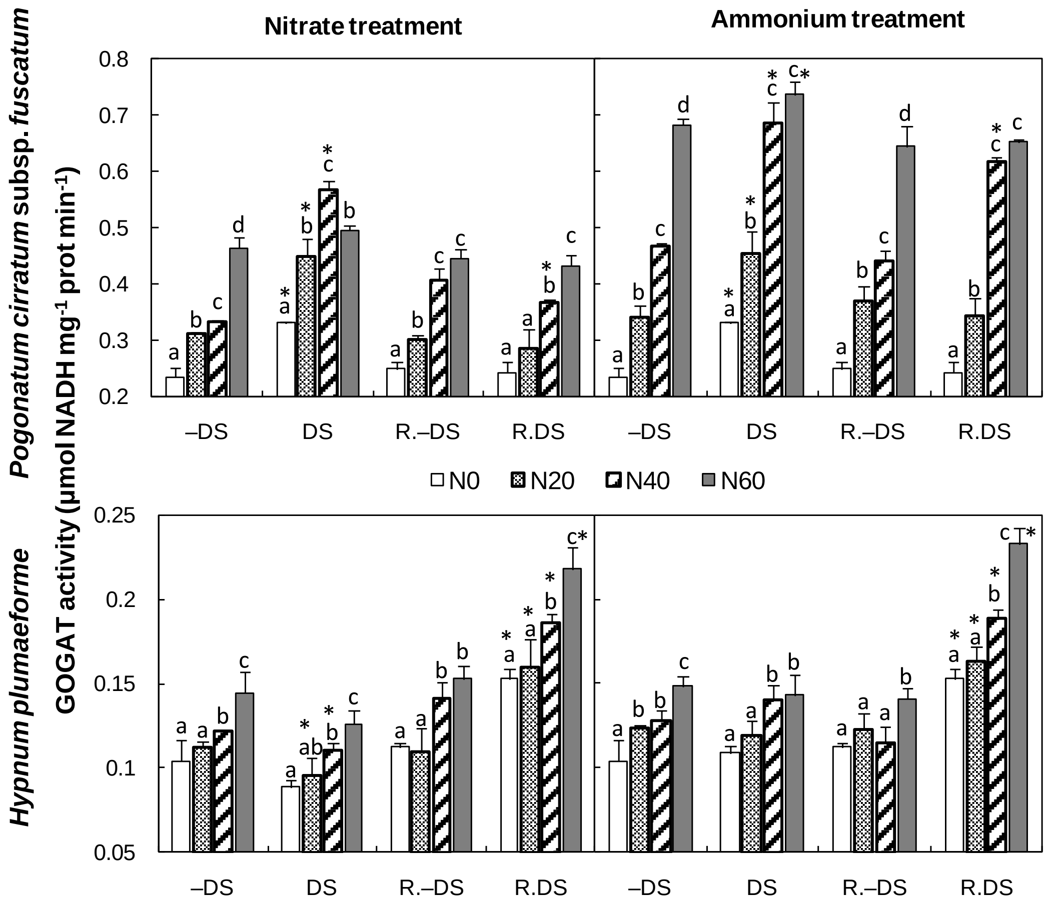

Figure S1 Glutamate synthase (GOGAT) activity in *Pogonatum cirratum* subsp. *fuscatum* and *Hypnum plumaeforme* exposed to indicated N treatments with or without water deficit stress (DS and –DS, respectively), and after a 10-day-recovery period from the treatments (R.DS and R.–DS, respectively). N0, N20, N40 and N60 indicate N supply levels of 0, 20, 40 and 60 kg N hm^{-2} yr^{-1}, respectively. Data presented as "means + S.D." (n=3). Different letters above the bars indicate significant differences between N concentrations ($p < 0.05$, one-way ANOVA, LSD test). Asterisks (*) above the DS and R.DS bars indicate significant differences between DS and –DS, R.DS and R.–DS respectively ($p < 0.05$, t-test).
